# Functional Role of Cerebellar Gamma Frequency in Motor Sequences Learning: a tACS Study

**DOI:** 10.1007/s12311-021-01255-6

**Published:** 2021-04-06

**Authors:** A. Giustiniani, V. Tarantino, M. Bracco, R. E. Bonaventura, M. Oliveri

**Affiliations:** 1 NEUROFARBA Department, University of Firenze, 50139 Firenze, Italy; 2 IRCCS San Camillo Hospital, 30126 Venezia, Italy; 3 Department of Psychology, Educational Science and Human Movement, University of Palermo, Viale delle Scienze, Edificio 15, 90128 Palermo, Italy; 4 Centre for Cognitive Neuroimaging, Institute of Neuroscience and Psychology, University of Glasgow, Glasgow, G12 8QB UK; 5 NeuroTeam Life and Science, Via Libertà 112, 90144 Palermo, Italy

**Keywords:** Transcranial alternating current stimulation (tACS), Transcranial magnetic stimulation (TMS), Serial reaction time task (SRTT), Implicit motor learning, Cerebellar stimulation

## Abstract

Although the role of the cerebellum in motor sequences learning is widely established, the specific function of its gamma oscillatory activity still remains unclear. In the present study, gamma (50 Hz)—or delta (1 Hz)—transcranial alternating current stimulation (tACS) was applied to the right cerebellar cortex while participants performed an implicit serial reaction time task (SRTT) with their right hand. The task required the execution of motor sequences simultaneously with the presentation of a series of visual stimuli. The same sequence was repeated across multiple task blocks (from blocks 2 to 5 and from blocks 7 to 8), whereas in other blocks, new/pseudorandom sequences were reproduced (blocks 1 and 6). Task performance was examined before and during tACS. To test possible after-effects of cerebellar tACS on the contralateral primary motor cortex (M1), corticospinal excitability was assessed by examining the amplitude of motor potentials (MEP) evoked by single-pulse transcranial magnetic stimulation (TMS). Compared with delta stimulation, gamma-tACS applied during the SRTT impaired participants’ performance in blocks where the same motor sequence was repeated but not in blocks where the new pseudorandom sequences were presented. Noteworthy, the later assessed corticospinal excitability was not affected. These results suggest that cerebellar gamma oscillations mediate the implicit acquisition of motor sequences but do not affect task execution itself. Overall, this study provides evidence of a specific role of cerebellar gamma oscillatory activity in implicit motor learning.

**Supplementary Information:**

The online version contains supplementary material available at 10.1007/s12311-021-01255-6.

## Introduction

Implicit motor learning refers to the acquisition of motor skills following repetition and without conscious awareness [1, 2]. Several studies report that a wide network of regions operates during motor skill learning, including the cerebellum, the prefrontal and primary motor cortex (M1), and the basal-ganglia [3]. Within this network, each region plays a unique role in the formation of new skills. In particular, the cerebellum has a key role in the acquisition of new motor skills as well as in the timing of motor sequences [4–6]. Indeed, cerebellar lesions impair the acquisition of sequences of movements [7, 8]. Likewise, repetitive transcranial magnetic stimulation (rTMS) and anodal transcranial direct current stimulation (tDCS) can interfere with cerebellar activity and affect motor-learning. Specifically, low-frequency cerebellar TMS, immediately before the execution of a serial reaction time task (SRTT), is able to disrupt implicit motor sequences learning (i.e., increasing reaction times) whereas anodal cerebellar tDCS can improve the performance on a similar task (i.e., reducing reaction times) [9, 10].

Although the role of the cerebellum is well established, the oscillatory dynamics mediating the acquisition of motor skills remain still to be clarified. For instance, 50 Hz transcranial alternating current stimulation (tACS) applied over the cerebellum improves the performance in a visuomotor task. Furthermore, the 50 Hz tACS induced a post-stimulation increase of M1 excitability [11] suggesting that cerebellum and M1 may communicate via gamma oscillations. Likewise, a weakening of cerebellar brain inhibition (CBI) was found. Indeed, the cerebellum exerts an inhibitory tone on the primary motor cortex (M1) so that applying magnetic or electric currents over the cerebellum induces secondary changes in motor cortex excitability levels [12, 13].

In line with these findings, high gamma frequency tACS (i.e., 70 Hz) delivered through electrodes placed simultaneously over the left cerebellar hemisphere and the right M1 has shown to improve motor performance in a force task requiring to track the movement of a target on a screen with the index finger [14]. The authors suggested that this improvement may reflect the strengthening of the synchronization between cerebellum and M1.

In a previous study, we found that M1 gamma-tACS (50 Hz) modulates the retrieval, but not the acquisition of a previously learned motor sequence in the SRTT. In addition, we found a reduction of M1 excitability after tACS [15]. An unexplored question, though, is whether cerebellar gamma activity also mediates implicit motor sequence learning. In the present study, we wanted to disentangle the role of M1 and cerebellum within the learning of a sequence of movements. We, therefore, applied a gamma-tACS (50 Hz) over the cerebellum while participants executed a SRTT. To control for frequency-specific effects and avoid entrainment of gamma harmonics and subharmonics, a delta (1 Hz) stimulation was applied as control condition [15, 16]. As both the stimulation frequencies may potentially induce an effect, to avoid misinterpretation of the results, we additionally compared performances during gamma and delta tACS with the performance of a group of participants that underwent sham tACS in a previous study conducted in our lab, with identical task and procedure.

Moreover, long-range after-effects on M1 were assessed by measuring MEP following cerebellar tACS. We hypothesized that if cerebellar gamma oscillations mediated implicit motor sequences learning [6], then we would expect an effect on task performance when the repetition of the same motor sequence is required. Moreover, if cerebellar tACS induced long-term effects on M1 activity, then we would also observe a modulation of corticospinal excitability, as indexed by MEP amplitude.

## Material and Methods

### Participants

Eighteen healthy right-handed volunteers (7 male, mean age 25.2 ± 4.1 years) took part in the experiment, after giving written informed consent. All participants were right-handed according to the Edinburgh handedness inventory [17] and naïve to the experimental hypotheses. Exclusion criteria were brain injury, neurological or psychiatric disorder, not-corrected vision deficits, intracranial metallic plates, cardiac pacemakers, pregnancy, family, or personal history of epilepsy. The study was approved by the ethical committee of the University Hospital “Paolo Giaccone” of Palermo and was conducted according to the declaration of Helsinki.

### Procedure

**Fig. 1:**
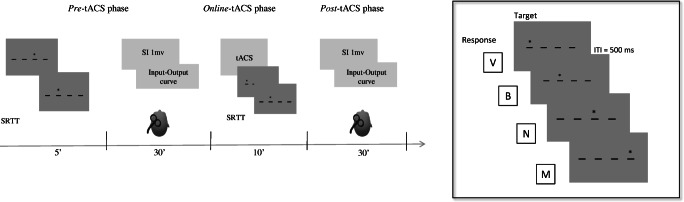
The figure shows the experimental procedure (left side) and the task’s structure (right side). In the *pre-*tACS phase, participants first performed the serial reaction time task (SRTT), followed by the assessment of the SI-1 mV (i.e., the lowest stimulus intensity needed to elicit MEP of 1 mV peak-to-peak) and of the input-output curve. In the *online*-tACS phase, stimulation was delivered during the execution of the SRTT (5 min). The electrodes were placed over the right cerebellum and the ipsilateral buccinator muscle. In the *post*-tACS phase, SI 1mV and the input-output procedure were again assessed. The panel on the right side shows the SRTT. The asterisk appeared in one of four possible positions marked by low dashes and corresponding to a key on a keyboard (V, B, N, M). Subjects responded to each asterisk by pressing with their right hand (index, middle, ring, pinkie) the key on the keyboard. Five hundred milliseconds after each response, the asterisk appeared at a new location. The task counted eight blocks, the same sequence was repeated in each block with the exception of blocks 1 and 6 were asterisks’ locations were randomly distributed

Participants took part in two experimental sessions, one for each stimulation frequency (1 Hz or 50 Hz), performed at least 48 h apart in a counterbalanced order. During the first session, participants familiarized with the experimental environment, underwent a brief interview about their medical history, and filled out the handedness inventory questionnaire [17]. During the experimental session, participants sat on a comfortable chair in front of a computer screen. Participants first executed the SRTT (*pre*-tACS phase). Soon after, the stimulation site and the motor threshold (MT) were identified, and 50 baseline MEP were collected through the input-output procedure. Afterward, participants executed a new version of the SRTT and simultaneously received tACS (*online*-tACS phase). Immediately after tACS (*post*-tACS phase), the TMS input-output procedure was replicated to test long-term changes in MEP amplitude (Fig. 1).

### Serial Reaction Time Task (SRTT)

SRTT is one of the most common methods used to assess implicit learning of motor sequences [18, 19]. In the present study, we used the same version as in Giustiniani et al. [15]. The task lasts 5 min and consisted of a motor cue (an asterisk of 1.2 cm diameter) appearing on a grey screen (17 inch, 1280 × 1024 pixel resolution) in one of four horizontally centred positions, marked by a low dash. Participants were instructed to press the key on a keyboard corresponding to the asterisk position as soon and as fast as they could by using one of the four fingers of the right hand (index, middle, ring, pinkie). The asterisk would remain on the screen until a response was given, then disappear and appear to the following position (Fig. 1). The inter-stimulus interval (the blank between asterisks) was fixed at 500 ms [20]. The task counted 8 blocks, interleaved with a self-paced break (few seconds to relax the hand). Participants were instructed to press any key to restart the task from the break. Each block, with the exclusion of blocks 1 and 6, included 6 repetitions of the same 12 motor cues/movement sequences. Blocks 1 and 6 presented, instead, a series of items in a pseudorandom order. Participants were unaware of the structure of task’s sequences. Four parallel versions of the SRTT were implemented, each with a different sequence. The interchangeability of these versions was verified in a pilot study. Each participant performed all the versions across the four time points (1 Hz pre-/post-tACS, 50 Hz pre-/post-tACS). Furthermore, during the first session, before the pre*-*tACS phase, two pseudorandom sequences of 12 stimuli each were administered for practice.

At the end of the experiment, participants were debriefed to make sure they were not aware of the repeating sequences. The qualitative debriefing consisted of a series of questions by which they were asked to report their perceived task difficulty and any other impression about the task, without mentioning the sequencing rules. None of the participant reported the sensation of a repeating sequence.

### Transcranial Alternating Current Stimulation (tACS)

TACS was delivered for the whole duration of the SRTT (online-tACS phase) through a BrainStim battery-driven electric stimulator (E.M.S., Bologna, Italy), connected with two conductive-rubber electrodes (5×5 cm^2^) placed in saline-soaked sponges. The centre of the active electrode was placed over the right cerebellar hemisphere (1 cm under and 3 cm right to the inion). The centre of the return electrode was placed over the ipsilateral buccinator muscle [21, 22], the impedance was kept below 10 kΩ. Stimulation intensity was set at 2 mA peak-to-peak corresponding to 0.08 mA/cm^2^ current density under each electrode, with a ramp up/down time of 30 s. Both participants and experimenters were blinded with respect to the stimulation frequency. At the end of each session, participants were asked about their perceived sensations during the stimulation using a structured questionnaire about tES-related sensations and discomforts [23]. We checked for the following sensations: itching, pain, burning, metallic/iron taste, warmth/ heat, fatigue, alertness, and others. Response options were none, mild, moderate, and strong. Most of the responses for all the sensations were either “none” or “mild” for both 1 and 50 Hz stimulation. None of the participants perceived phosphenes during the stimulation. Moreover, participants were not able to distinguish between the two stimulation frequencies. During the 1-Hz stimulation, two participants reported sensations of metallic taste and dizziness. For these participants, the stimulation was immediately interrupted, and they were excluded from the study and replaced by two additional participants (final *n* = 18).

### Transcranial Magnetic Stimulation (TMS)

Single-pulse TMS was delivered over the left M1 using a MagStim Super Rapid 2 biphasic magnetic stimulator (Magstim Company, Whiteland, Wales, UK) through a 70-mm figure-eight coil. MEP were recorded with surface Ag/AgCl electrodes placed over the first dorsal interosseous (FDI) muscle of the right hand, arranged in a belly-tendon montage, and connected to a stimulator-integrated EMG amplifier. Raw EMG signals were band-pass filtered (2 Hz to 10 kHz). The coil was placed tangentially to the scalp with the handle pointing in antero-medial orientation, 45° from the interhemispheric line of the participant’s head, so that the current flowed in a posterior to anterior direction inducing the strongest tissue current in the reversal phase of the pulse [24]. To define stimulation location, we first delivered TMS pulses over C3 (as in 10-20 EEG system) and surrounding sites, until we found the optimal site to elicit the largest evoked potential in the FDI muscle at rest. Coil position was then fixed and recorded through an optic tracking system (SoftTaxic Neuronavigator system; E.M.S., Bologna, Italy), which allowed the monitoring of the coil position throughout the whole session. TMS intensity was set relative to the lowest stimulus intensity (% maximum stimulator output) needed to elicit MEP of 1 mV peak-to-peak amplitude (SI-1mV) in at least three out of five pulses. Participants’ corticospinal excitability was assessed by using the input-output curve procedure. Namely, 50 peak-to-peak MEP were acquired at each stimulus intensity ranging from 100 to 140% of the SI-1mV, in steps of 10% (10 single pulses for each intensity delivered every 8–10 s), immediately before and after tACS [25, 26].

### Data Analysis

#### SRTT

We measured tACS modulatory effects on the SRTT by using participants’ response times (RTs). Inferential statistics were not computed for error rates because the task typically shows very high percentage of accurate responses [15]. RTs of incorrect responses and shorter than 100 ms were removed before the analysis (overall 3.6% of trials). To improve the normality distribution of RTs, a log-transformation was applied to raw RTs data (Kolmogorov-Smirnov test on raw RTs: D = 0.11, p < .001, skewness = 2.24; K-S test on log-RT: D = 0.04, p < .001, skewness = 0.58) [27]. Then, RTs were modelled by a multiple linear regression model. Namely, the following model (in R notation) was fitted by means of the *lme4* package [28]: RT ~ Stimulation frequency × Time × Block + (1 | ID), including stimulation frequency (delta vs. gamma stimulation), time (pre- vs. online-tACS phase), and block (1–8) as predictors. Delta stimulation, pre-tACS, and block 1 was entered as reference levels (set to 0). Block number was considered as a factor as blocks qualitatively differed: the same motor sequence was repeated across blocks 2, 3, 4, 5, 7, and 8, whereas new/pseudorandom sequences were presented in blocks 1 and 6. Motor performance was expected to improve across the blocks containing the repeated sequence (i.e., blocks 2–5, 7–8) and to worsen in the block containing pseudorandom sequences (i.e., block 6). Participant number (ID) was entered as random factor. To make sure participants learned the given sequence, in a separate regression model, we contrasted RTs in block 5, i.e., where the motor sequence was presented the maximal number of times, and thus, the amount of learning should be maximal, to RTs in block 6, i.e., containing a new/pseudorandom motor sequence, and thus, interrupting the learned sequence (RTs_Block5_ vs RTs_Block6_). This contrast represents an index of implicit learning [29]. The plotted residual values of all the models were normally distributed around zero (from max 0.5 to min -0.5).

#### Input-output curve

The tACS after-effect on corticospinal excitability was assessed by analysing the MEP input-output curve. Data were fitted with the following regression model: MEP amplitude ~ Stimulation frequency × Time × Pulse intensity + (1 | ID). Pulse intensity has five levels: 100, 110%, 120, 130, and 140%.

In all the regression models, *p*-values were estimated by means of the *lmerTest* package; the Satterthwaite’s approximation was applied for computing the degrees of freedom [30].

## Results

### SRTT

**Fig. 2:**
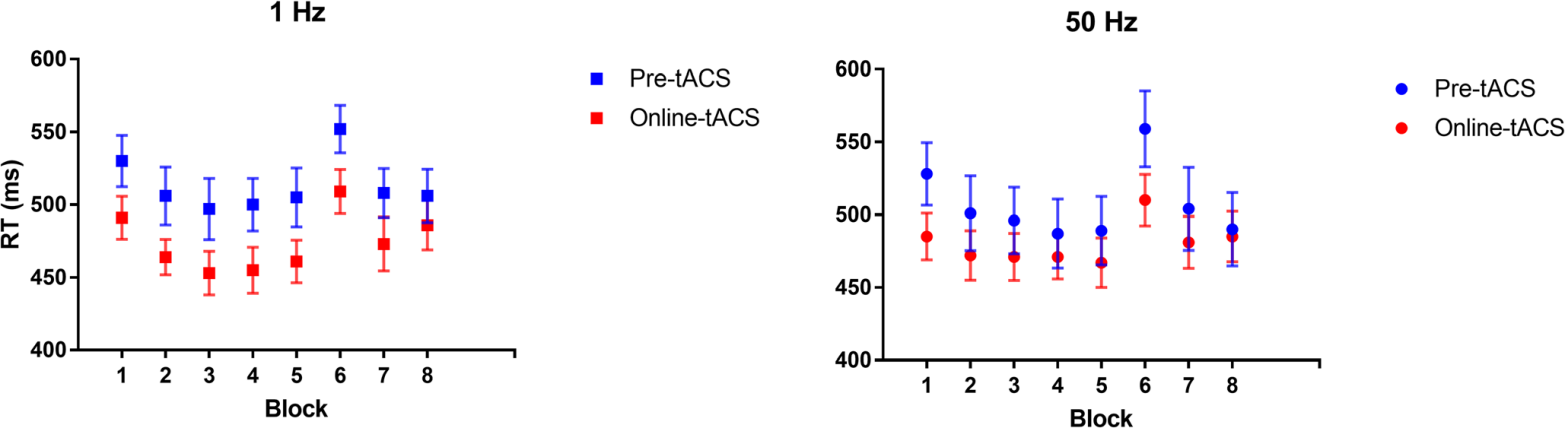
Mean response times (RTs) expressed in milliseconds (ms) across task blocks and stimulation frequency (Delta, 1 Hz vs. Gamma, 50 Hz), before (pre) or during (online) tACS. The repeating sequence was presented from block 2 to block 5 and in blocks 7 and 8, respectively. A random sequence was embedded in blocks 1 and 6. Error bars represent standard deviation of mean RT

**Fig. 3:**
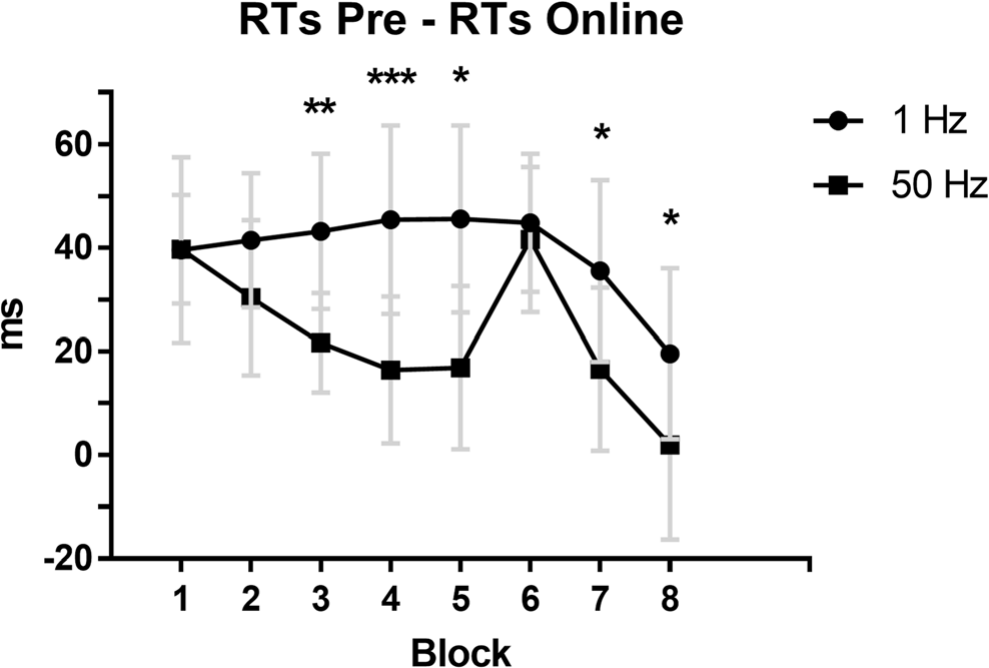
Difference between mean response times (RTs) in the pre*-*tACS phase and mean RTs in the online-tACS phase across task blocks and stimulation types (Delta, 1 Hz vs. Gamma, 50 Hz). Error bars represent standard error of mean RTs difference. Here, RTs are expressed in milliseconds (ms) whereas statistical analyses were performed on log-transformed data, baseline corrected to block 1

Overall, a mean accuracy of 97% (SD = 18) in both the pre- and online 1 Hz sessions and a mean accuracy of 97% (SD = 16) in both the pre- and online 50 Hz sessions was obtained. The mean RTs across blocks and tACS sessions are plotted in Fig. 2. A main effect of time and a main effect of block were found. Namely, participants were overall significantly faster in the online-tACS phase relative to the pre-tACS phase (*t* = −6.78, *p* < .001), and in all blocks relative to block 1 (blocks 2–5, 7–8: *ts* < −4.64, *ps* < .001), but block 6 (*t* = 3.21, *p* = .001). No main effect of Stimulation frequency was found (*t* = -.34, *p* = .734). Importantly, the Stimulation frequency × Time × Block interaction revealed that the RTs change from pre- to online-tACS phase was smaller when gamma stimulation was applied relative to delta stimulation, in blocks 3, 4, 5, 7, and 8 (*ts* > 2.02, *ps* ≤ .05; Fig. 3). In other words, participants became significantly faster during the delta stimulation compared with the gamma stimulation. On the other hand, RTs on block 6 were not affected by Stimulation frequency × Time (*t* = 0.12, *p* = 0.9).

When we contrasted the RTs in block 5 to RTs in block 6 (learning index) in the online tACS phase, a significant Stimulation frequency × Time interaction emerged (*t* = -2.29, *p* = .022), which showed that the difference between RTs in block 5 and RTs in block 6 was larger during the delta stimulation relative to the gamma stimulation.

The effect on the learning index was similar to that previously observed during the sham tACS in a previous study of our lab with identical task and design ([15], Table 4S and Fig. 1S). Although this comparison should be taken cautiously since the data refer to an independent study, we merged the data of the two experiments and statistically tested the effects of Stimulation frequency (1 Hz vs. 50 Hz vs. Sham), Time (pre vs. online), and block (5 vs. 6) on RTs. The analysis confirmed the presence of a three-way interaction, that is, the RT difference between block 6 and block 5 in the online-tACS phase relative to the pre-tACS phase was smaller in the 50 Hz condition compared with the Sham (*t* = −2.88, *p* = .004), but it did not differ between the 1 Hz and the Sham session (*t* = −.7, *p* = .485).

To check for carry-over effects and differences at baseline (pre-tACS), we assessed the effect of the stimulation order using the following model: RT ~ Stimulation frequency × session + (1 | ID). The statistical analysis revealed no differences between the pre-tACS in the two stimulation conditions (*t*s < .043, *p*s > .372).

### Input-output curve

**Fig. 4:**
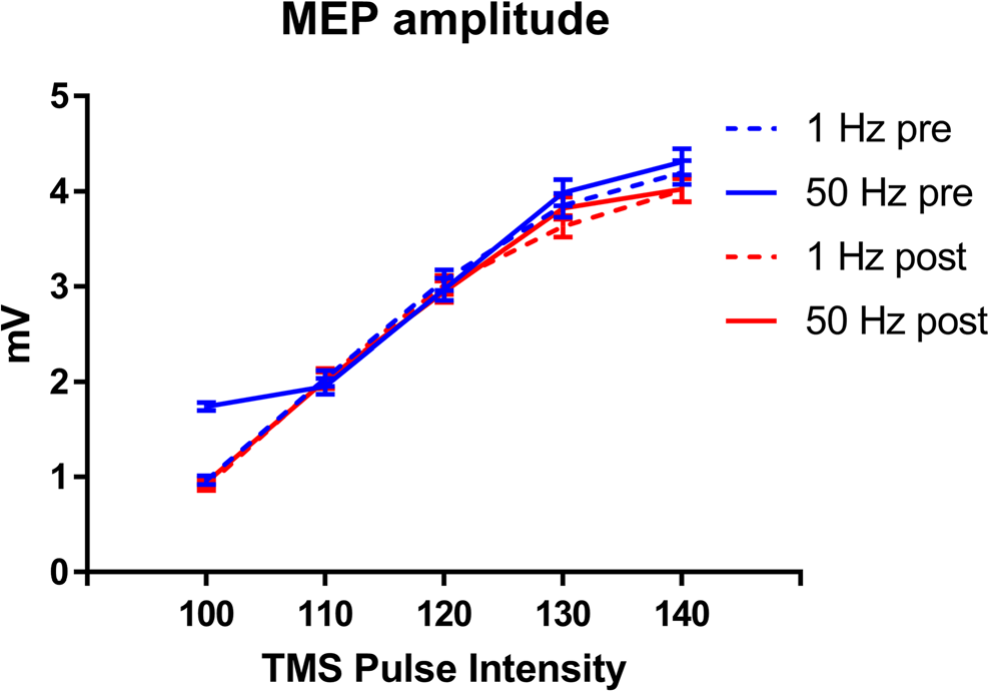
Mean amplitude of motor-evoked potential (MEP) in pre- and post-tACS phases of the two stimulation frequencies (Delta, 1 Hz vs. Gamma, 50 Hz) across transcranial magnetic stimulation (TMS) pulse intensities (from 100 to 140% of the TMS intensity that elicited a 1 mV peak-to-peak MEP, SI-1mV). Error bars represent standard error of mean MEP amplitude

Figure 4 depicts the mean MEP amplitude evoked by single-pulse TMS at varying intensities after tACS. As expected, MEPs amplitude significantly increased with increasing TMS intensities (*t* = 18.7, *p* < .001). However, neither the effect of Stimulation frequency (t = -0.17, *p* = .867) nor the Time (*t* = -0.6, *p* = .55) and the interactions (ts < 0.75, *p*s > 0.454) were significant.

All the complete regression models are reported in the Supplementary materials.

## Discussion

The aim of the present study was to assess the role of cerebellar gamma oscillations in motor sequences learning. To this aim, during the execution of an implicit motor learning task (SRTT), we delivered either gamma—or delta—tACS over the cerebellar hemisphere ipsilateral to the performing hand. Compared with delta tACS, we found that, during the gamma-tACS, participants did not show an increased performance in blocks where the same motor sequence was repeated over time and the motor performance was expected to maximally improve. Interestingly, no differences between the two stimulation frequencies emerged during the execution of the new/pseudorandom motor sequence. This finding was confirmed by a reduced difference between the block where we expected the sequence being maximally learned due to repetition (i.e., block 5) and the block where the new/pseudorandom sequence was presented and learning was interfered (i.e., block 6) during gamma—compared with delta—tACS.

The role of the cerebellum is principally to create predictions and internal models of the external stimuli [31]. These models are used to fast detect future regularities and deviances (errors) so that learning can take place and motor responses can be optimized. Therefore, implicit motor learning, such as the one we observed during the execution of SRTT, is indexed by a progressive reduction of RTs when stimuli/movements follow a fixed predictable order (regular motor sequences) [32]. In the present study, during the gamma stimulation, the progressive RTs reduction that usually emerges over time during learning of fixed sequences was missing, so that the performance reflected a flattened learning curve across blocks (see Fig. 3). We speculate that this result was due to interference caused by cerebellar gamma-tACS on the formation of internal models, which, in turn, resulted in a disrupted learning of the motor sequence [33, 34].

In a previous study [15], we applied a gamma-tACS over M1 during the execution of the same motor sequence task, and observed a disruption of participants’ performance only in the two last task blocks (blocks 7 and 8). We interpreted this result as due to gamma-tACS impairing participants’ ability to retrieve the previously learned motor sequence. By comparing these findings to the present results, we may speculate that while gamma-tACS over the cerebellum interferes with the acquisition phase of a motor sequence (blocks 3–5) and, consequently, with its retrieval at later stages (blocks 7–8), gamma-tACS over M1 interferes with retrieval of previously learned sequential movements (blocks 7–8 only), without affecting the initial acquisition of motor traces. The present findings are consistent with previous imaging studies demonstrating the cerebellum having a key role on the formation of implicit motor skills [3, 35], and M1 playing a role during relatively later stages, such as the retention phase [3, 33–35]. Furthermore, these findings are in line with a previous tDCS study that dissociated the contribution of cerebellum in the acquisition and of M1 in the retention of motor memories [36].

To the best of our knowledge, this is the first evidence showing a causal role of cerebellar gamma oscillations in the acquisition of implicit motor sequences. A recent study investigated the role of higher gamma frequency band in motor memory showing that 70 Hz tACS enhances retention in a task requiring to explicitly memorize fingers’ movements, needed to track a visual target on a screen [15]. Indeed, a performance’s improvement was observed 1 day after stimulation. Conversely, no changes in performance were found during tACS. Remarkably, in this study, the authors compared two different tACS montages (cerebellum only vs. cerebellum and M1) reporting no effects when tACS electrodes were placed over the cerebellum only. Overall, the authors conclude that synchronizing the activity of cortical and cerebellar areas in the gamma frequency band affects explicit motor learning over a wide intervals of time (i.e., 1 day after learning). In a more recent study, 50 Hz left cerebellar tACS did not affect performance in an explicit motor learning task requiring the execution of a grip force to move a cursor [37]. The difference in montage and hand between this study (left cerebellum and non-dominant hand) and our study (right cerebellum and right hand) might account for the divergent results and suggests that the effects depend on the stimulated hemisphere. Moreover, the divergent results might depend on the specific effect of 50 Hz gamma on implicit (our study) rather than explicit (Wessel’s and Miyaguchy’s studies) motor learning.

At a first glance, the impaired rather than the improved performance induced by gamma-tACS may appear inconsistent with the evidence of gamma synchronization in a wide network of brain regions during motor tasks [38]. However, one should consider that motor learning processes relative to cerebellar activity depend on the combined activity of Purkinje cells and parallel fibers [39]. Together they are responsible of cerebellar long-term depression (LTD) plasticity, which, we know, occurs during motor sequence learning [40]. Due to the low intensity of the stimulation, we may speculate that alternating currents reached the more external layer of cerebellum where Purkinje cells have their bodies [41]. By modulating the activity of these cells in the cerebellar cortex, gamma-tACS might have prevented the LTD processes needed for the formation of internal models and consequently for sequence learning [42, 43]. Indeed, inducing gamma oscillations might have led to long-term potentiation (LTP) [44].

On the other hand, as Purkinje cells exhibit activity at 50 Hz [44], we cannot exclude that the observed interference reflected an U-shape dose-effect [45]. Indeed, when an optimal level of oscillatory activity is reached, an increase of the power of that specific brain rhythm would deteriorate performance [46, 47]. In this case, endogenous cerebellar gamma oscillations could have reached high levels of power during the task. Therefore, externally applying gamma oscillatory currents could have perturbed cells activity by inducing homeostatic plasticity and prevented cells’ normal functioning during the formation of the motor sequence.

Finally, another possible explanation for the disruptive effect is that suboptimal components of the gamma frequency band have been entrained here. Indeed, Purkinje cell’s simple spikes are distributed in a wider range of frequencies, including the higher gamma band [48].

We did not find changes in corticospinal excitability measured after the end of the stimulation; therefore we might conclude that gamma-tACS specifically impaired cerebellar functioning and procedural learning components, probably without affecting M1 excitability. However, one might argue that some changes could have occurred on M1 excitability during tACS (online effect), which did not survive beyond the end of stimulation (after effect). Furthermore, input-output curve is a measure of cortical excitability; thus, we cannot exclude that some changes occurred in terms of cortical inhibition and that other methods (i.e., short or long intracortical inhibition) might have been more sensitive in detecting these changes. Our results are in contrast with a previous study of Naro et al. [11] reporting changes in cortical excitability (i.e., MEP amplitude) after 50 Hz cerebellar tACS. Nevertheless, methodological differences might account for this difference, such as the tACS duration (1 min in the Naro’s study vs. the whole task duration in the present study), the procedure used to test corticospinal excitability (TMS-MEP vs. TMS input-output curve), and the brain state during tACS (resting vs. task execution). Further studies are needed to explore the effect of cerebellar tACS on corticospinal excitability, taking into account the ongoing brain state (state dependency effect). A limitation of the study that we should acknowledge is that assessing paired-pulse cerebellar inhibition might have been a more sensitive measure to evaluate the impact of tACS on the cerebello-M1 tract. Additionally, throughout the input-output procedure, we collected only 10 MEP for each stimulation intensity, assessing MEP amplitude in 20–30 trials might have ensured a more stable and reliable measure of M1 excitability [49].With respect to tACS, another limitation of the study is the poor focality of stimulation. Indeed, given the montage and the electrodes’ size, we cannot exclude a spread of the current over other cortical areas [50]. The future use of high-density montages would elucidate this issue.

## Conclusions

The present study suggests that gamma-tACS on cerebellum perturbs the acquisition of motor sequences. This impairment of cerebellar activity during gamma-tACS does not induce long-lasting effects on corticospinal excitability measured with the input-output procedure. These results confirm previous studies showing the pivotal role of the cerebellum in procedural learning and add new evidence on the specific contribution of cerebellar gamma oscillations, encouraging future studies to explore the specific functional role of gamma cerebellar oscillatory activity in motor as well as in cognitive learning.

## Supplementary Information

ESM 1 (DOCX 152 kb)
