# Supplementary material for: Functional Role of Cerebellar Gamma Frequency in Motor Sequences Learning: a tACS Study
Source: Cerebellum. 2021 Apr 6;20(6):913–21. doi: 10.1007/s12311-021-01255-6 (PMC8674154; doi:10.1007/s12311-021-01255-6)
Supplement: Supplementary file 1 — (DOCX 152 kb) [file 12311_2021_1255_MOESM1_ESM.docx]

**Functional role of cerebellar gamma frequency in** **motor sequences learning: a tACS study**

Giustiniani A, Tarantino V., Bracco M., Bonaventura R.E.,Oliveri M.

Supplementary tables

**Table 1S.** Results of the linear mixed-effects model fitted to log-transformed response times (RTs) implemented by the *lmer* function (lme4 R package [1]). Model: RT ∼ Stimulation Frequency × Time × Block + (1 | ID). Estimates = beta coefficients; CI = confidence interval; *p*-values were computed by the *lmerTest* package [2,3]. Fixed factors: Stimulation frequency (delta vs. gamma), Time (pre- vs. online-tACS), Block (1 vs. 2,3,4,5,6,7,8). Random factor: ID (participant identification number). σ^2^ = within-group (or residual) variance; τ_00_ = between-group random intercept variance; ICC = Intraclass Correlation Coefficient; Marginal R^2^ = variance explained by the fixed effects; Conditional R^2^ = variance explained by the fixed and mixed effects.

|  | **RTs** | | | |
| --- | --- | --- | --- | --- |
| *Predictors* | *Estimates* | *CI* | *t test* | *p* |
| (Intercept) | 2.71 | 2.68 – 2.74 | 177.355 | **<0.001** |
| Stimulation frequency [gamma] | -0.00 | -0.01 – 0.01 | -0.340 | 0.734 |
| Time [online] | -0.03 | -0.04 – -0.02 | -6.785 | **<0.001** |
| Block [Block2] | -0.02 | -0.03 – -0.01 | -4.640 | **<0.001** |
| Block [Block3] | -0.03 | -0.04 – -0.02 | -6.689 | **<0.001** |
| Block [Block4] | -0.03 | -0.04 – -0.02 | -6.793 | **<0.001** |
| Block [Block5] | -0.03 | -0.04 – -0.02 | -6.364 | **<0.001** |
| Block [Block6] | 0.01 | 0.01 – 0.02 | 3.206 | **0.001** |
| Block [Block7] | -0.02 | -0.03 – -0.01 | -4.810 | **<0.001** |
| Block [Block8] | -0.03 | -0.03 – -0.02 | -6.012 | **<0.001** |
| Stimulation frequency [gamma] × Time [online] | 0.00 | -0.01 – 0.01 | 0.453 | 0.651 |
| Stimulation frequency [gamma] × Block [Block2] | -0.01 | -0.02 – 0.01 | -0.831 | 0.406 |
| Stimulation frequency [gamma] × Block [Block3] | -0.00 | -0.01 – 0.01 | -0.234 | 0.815 |
| Stimulation frequency [gamma] × Block [Block4] | -0.01 | -0.02 – 0.00 | -1.515 | 0.130 |
| Stimulation frequency [gamma] × Block [Block5] | -0.01 | -0.02 – 0.00 | -1.793 | 0.073 |
| Stimulation frequency [gamma] × Block [Block6] | 0.01 | -0.01 – 0.02 | 0.831 | 0.406 |
| Stimulation frequency [gamma] × Block [Block7] | -0.01 | -0.02 – 0.00 | -1.287 | 0.198 |
| Stimulation frequency [gamma] × Block [Block8] | -0.01 | -0.03 – -0.00 | -2.289 | **0.022** |
| Time [online] × Block [Block2] | -0.00 | -0.01 – 0.01 | -0.346 | 0.730 |
| Time [online] × Block [Block3] | -0.01 | -0.02 – 0.01 | -1.058 | 0.290 |
| Time [online] × Block [Block4] | -0.01 | -0.02 – 0.00 | -1.541 | 0.123 |
| Time [online] × Block [Block5] | -0.00 | -0.02 – 0.01 | -0.608 | 0.543 |
| Time [online] × Block [Block6] | -0.00 | -0.01 – 0.01 | -0.368 | 0.713 |
| Time [online] × Block [Block7] | 0.00 | -0.01 – 0.01 | 0.169 | 0.866 |
| Time [online] × Block [Block8] | 0.01 | 0.00 – 0.02 | 2.013 | **0.044** |
| (Stimulation frequency [gamma] × Time [online]) × block [Block2] | 0.01 | -0.00 – 0.03 | 1.685 | 0.092 |
| (Stimulation frequency [gamma] × Time [online]) × block [Block3] | 0.02 | 0.01 – 0.04 | 2.655 | **0.008** |
| (Stimulation frequency [gamma] × Time [online]) × block [Block4] | 0.03 | 0.02 – 0.05 | 3.731 | **<0.001** |
| (Stimulation frequency [gamma] × Time [online]) × block [Block5] | 0.02 | 0.00 – 0.04 | 2.474 | **0.013** |
| (Stimulation frequency [gamma] × Time [online]) × block [Block6] | 0.00 | -0.02 – 0.02 | 0.121 | 0.904 |
| (Stimulation frequency [gamma] × Time [online]) × block [Block7] | 0.02 | 0.00 – 0.04 | 2.235 | **0.025** |
| (Stimulation frequency [gamma] × Time [online]) × block [Block8] | 0.02 | 0.00 – 0.03 | 2.023 | **0.043** |
| Random Effects | | | | |
| σ^2^ | 0.01 | | | |
| τ_00_ _ID_ | 0.00 | | | |
| ICC | 0.25 | | | |
| N _ID_ | 18 | | | |
| Observations | 39701 | | | |
| Marginal R^2^ / Conditional R^2^ | 0.024 / 0.272 | | | |

**Table 2S.** Results of the linear mixed-effects model fitted to log-transformed response times (RTs) by the *lmer* function (lme4 R package [1]). Model: RT ∼ Stimulation Frequency × Time × Block + (1 | ID). Estimates = beta coefficients; CI = confidence interval. Fixed factors: Stimulation frequency (delta vs. gamma), Time (pre- vs. online-tACS), Block (5 vs. 6). Random factor: ID (participant identification number). σ^2^ = within-group (or residual) variance; τ_00_ = between-group random intercept variance; ICC = Intraclass Correlation Coefficient; Marginal R^2^ = variance explained by the fixed effects; Conditional R^2^ = variance explained by the fixed and mixed effects.

|  | **RTs** | | | |
| --- | --- | --- | --- | --- |
| *Predictors* | *Estimates* | *CI* | *t test* | *p* |
| (Intercept) | 2.68 | 2.65 – 2.71 | 168.930 | **<0.001** |
| Stimulation frequency [gamma] | -0.01 | -0.02 – -0.00 | -2.814 | **0.005** |
| Time [online] | -0.03 | -0.04 – -0.02 | -7.424 | **<0.001** |
| Block [Block6] | 0.04 | 0.03 – 0.05 | 9.254 | **<0.001** |
| Stimulation frequency [gamma] × Time [online] | 0.03 | 0.01 – 0.04 | 4.079 | **<0.001** |
| Stimulation frequency [gamma] × Block [Block6] | 0.02 | 0.00 – 0.03 | 2.513 | **0.012** |
| Time [online] × Block [Block6] | 0.00 | -0.01 – 0.01 | 0.225 | 0.822 |
| (Stimulation frequency [gamma] × Time [online]) × Block [Block6] | -0.02 | -0.04 – -0.00 | -2.286 | **0.022** |
| Random Effects | | | | |
| σ^2^ | 0.01 | | | |
| τ_00_ _ID_ | 0.00 | | | |
| ICC | 0.26 | | | |
| N _ID_ | 18 | | | |
| Observations | 9858 | | | |
| Marginal R^2^ / Conditional R^2^ | 0.040 / 0.286 | | | |

**Table 3S.** Results of the linear mixed-effects model fitted to amplitude of motor-evoked potentials (MEP). Model: MEP ∼ Stimulation Frequency × Time × TMS intensity + (1 | ID). Estimates = beta coefficients; CI = confidence interval. Fixed factors: Stimulation frequency (delta vs. gamma), Time (pre- vs. online-tACS), TMS intensity (100 vs. 110, 120, 130, 140 %). Random factor: ID (participant identification number). σ^2^ = within-group (or residual) variance; τ_00_ = between-group random intercept variance; ICC = Intraclass Correlation Coefficient; Marginal R^2^ = variance explained by the fixed effects; Conditional R^2^ = variance explained by the fixed and mixed effects.

|  | **MEP** | | | |
| --- | --- | --- | --- | --- |
| *Predictors* | *Estimates* | *CI* | *t test* | *p* |
| (Intercept) | -7.10 | -8.20 – 6.00 | -12.648 | **<0.001** |
| Stimulation frequency [gamma] | -0.63 | -2.03 – -0.78 | -0.873 | 0.383 |
| Time [post] | 0.43 | -0.98 – -1.83 | 0.597 | <0.550 |
| TMS intensity | 0.08 | 0.07 – 0.09 | 19.730 | **<0.001** |
| Stimulation frequency [gamma] × Time [post] | 0.51 | 1.48 – 2.49 | 0.499 | 0.618 |
| Stimulation frequency [gamma] × TMS intensity | 0.01 | 0.01 – 0.02 | 0.886 | 0.375 |
| Time [post] × TMS intensity | -0.00 | -0.02 – 0.01 | -0.749 | 0.454 |
| (Stimulation frequency [gamma] × Time [post]) × TMS intensity] | -0.00 | -0.02 – -0.01 | -0.477 | 0.633 |
| Random Effects | | | | |
| σ^2^ | 3.16 | | | |
| τ_00_ _ID_ | 1.05 | | | |
| ICC | 0.25 | | | |
| N _ID_ | 18 | | | |
| Observations | 3599 | | | |
| Marginal R^2^ / Conditional R^2^ | 0.243 / 0.432 | | | |

**Table 4S.**  Results of the linear mixed-effects model that contrasted Block 5 to Block 6 (learning index [4]) in sham [5], delta, and gamma conditions. Model: RT ∼ Stimulation Frequency × Time × Block + (1 | ID). Estimates = beta coefficients; CI = confidence interval. Fixed factors: Stimulation frequency (sham vs. delta vs. gamma), Time (pre- vs. online-tACS), Block (5 vs. 6). Random factor: ID (participant identification number). σ^2^ = within-group (or residual) variance; τ_00_ = between-group random intercept variance; ICC = Intraclass Correlation Coefficient; Marginal R^2^ = variance explained by the fixed effects; Conditional R^2^ = variance explained by the fixed and mixed effects.

|  | **RT** | | | |
| --- | --- | --- | --- | --- |
| *Predictors* | *Estimates* | *CI* | *t test* | *p* |
| (Intercept) | 2.68 | 2.64 – 2.72 | 137.216 | **<0.001** |
| Stimulation frequency [delta] | 0.00 | -0.05 – -0.05 | 0.054 | 0.957 |
| Stimulation frequency [gamma] | -0.01 | -0.06 – -0.04 | -0.408 | 0.683 |
| Time [online] | -0.03 | -0.04 – -0.02 | -6.948 | **<0.001** |
| Block [6] | 0.05 | 0.04 – 0.05 | 9.519 | **<0.001** |
| Stimulation frequency [delta] × Time [online] | 0.00 | 0.01 – 0.01 | 0.072 | 0.943 |
| Stimulation frequency [gamma] × Time [online] | 0.03 | -0.01 – 0.04 | 3.954 | **<0.001** |
| Stimulation frequency [delta] × Block [6] | -0.00 | -0.02 – -0.01 | -0.516 | 0.606 |
| Stimulation frequency [gamma] × Block [6] | 0.01 | -0.00 – -0.03 | 1.908 | 0.056 |
| Time [online] × Block [6] | 0.01 | -0.01 – -0.02 | 1.172 | 0.241 |
| Stimulation frequency [delta] × Time [online] × Block [6] | -0.01 | -0.03 – -0.01 | -0.698 | 0.485 |
| Stimulation frequency [gamma] × Time [online] × Block [6] | -0.03 | -0.05 – -0.01 | -2.882 | **0.004** |
| Random Effects | | | | |
| σ^2^ | 0.01 | | | |
| τ_00_ _ID_ | 0.01 | | | |
| ICC | 0.32 | | | |
| N _ID_ | 35 | | | |
| Observations | 14342 | | | |
| Marginal R^2^ / Conditional R^2^ | 0.037 / 0.350 | | | |

Figure 1S. Mean response times (RTs) across task blocks before (pre) or during (online) Sham tACS (data from [4]). Error bars represent standard deviation of mean RTs.

**
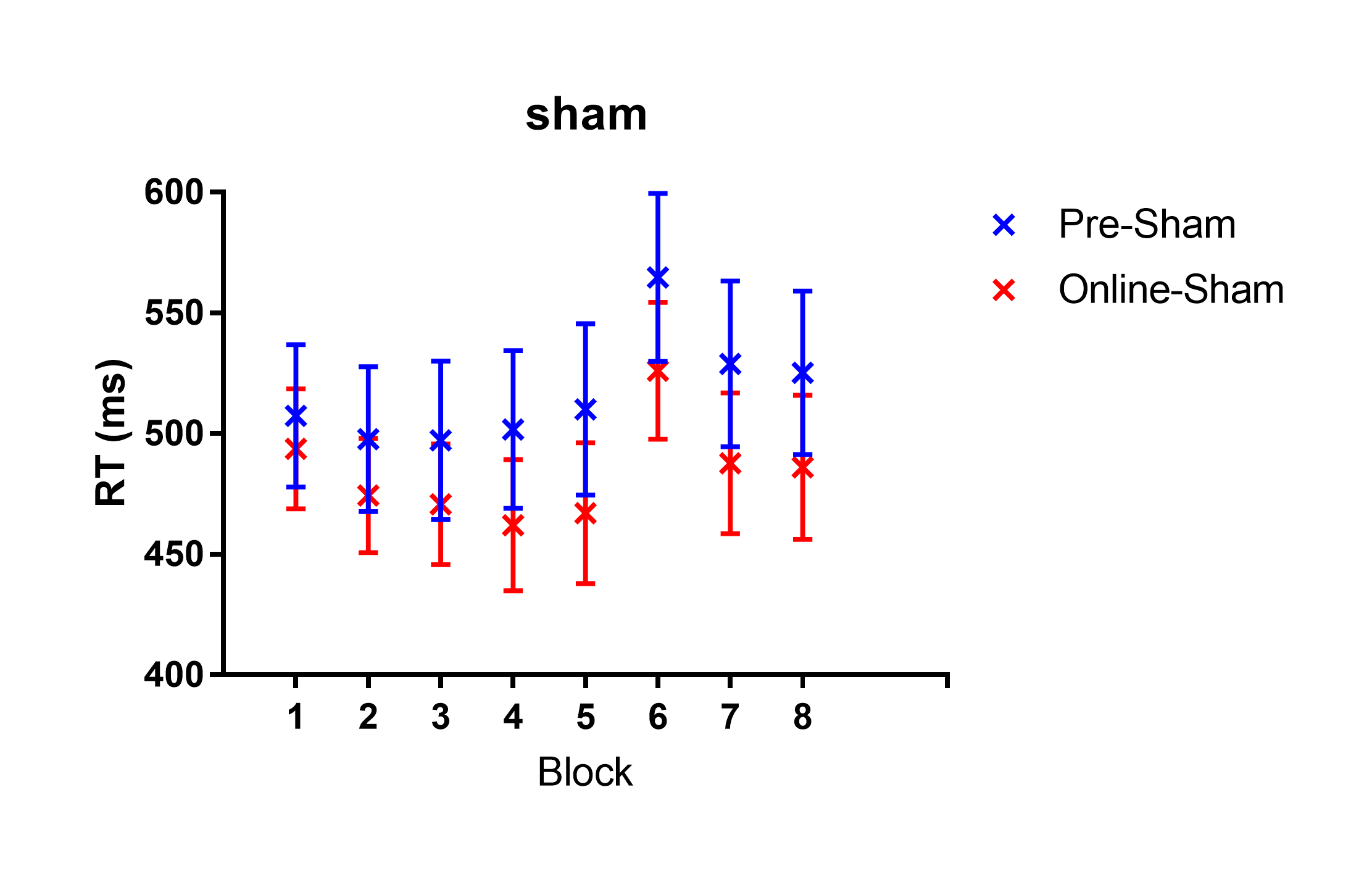
**

**References**

[1] D. Bates, M. Maechler, B. Bolker, S. Walker, Fitting linear miced-effects models using lme4, J. Stat. Softw. 67 (2015) 1–48. doi:10.18637/jss.v067.i01.

[2] S.G. Luke, Evaluating significance in linear mixed-effects models in R, Behav. Res. Methods. 49 (2017) 1494–1502. doi:10.3758/s13428-016-0809-y.

[3] A. Kuznetsova, P.B. Brockhoff, R.H.B. Christensen, lmerTest Package: Tests in Linear Mixed Effects Models , J. Stat. Softw. 82 (2017). doi:10.18637/jss.v082.i13.

[4] G.G. Ambrus, L. Chaieb, R. Stilling, H. Rothkegel, A. Antal, W. Paulus, Monitoring transcranial direct current stimulation induced changes in cortical excitability during the serial reaction time task, Neurosci. Lett. 616 (2016) 98–104. https://doi.org/10.1016/j.neulet.2016.01.039.

[5] A. Giustiniani, V. Tarantino, R.E. Bonaventura, D. Smirni, P. Turriziani, M. Oliveri, Effects of low-gamma tACS on primary motor cortex in implicit motor learning, Behav. Brain Res. 376 (2019)
